# Abnormal hippocampal miR-1b expression is ameliorated by regular treadmill exercise in the sleep-deprived female rats

**DOI:** 10.22038/ijbms.2019.31988.7734

**Published:** 2019-05

**Authors:** Lily Mohammadipoor-ghasemabad, Mohammad Hossein Sangtarash, Saeed Esmaeili-Mahani, Vahid Sheibani, Hosein Ali Sasan

**Affiliations:** 1 Department of Biology, Faculty of Science, University of Sistan and Baluchestan, Zahedan, Iran; 2 Laboratory of Molecular Neuroscience, Neuroscience Research Center, Institute of Neuropharmacology, Kerman University of Medical Science, Kerman, Iran; 3 Department of Biology, Faculty of Science, Shahid Bahonar University of Kerman, Kerman, Iran

**Keywords:** Female rats, Hippocampus, Mir-1b, Sleep deprivation, Treadmill training

## Abstract

**Objective(s):**

The protective effect of regular running on sleep deprivation (SD)-induced cognitive impairment has been revealed. In this study, we focused on the effects of regular exercise, sleep deprivation and both of them together on the microRNA-1b (miR-1b) expression and their relation to the behavioral parameters and brain-derived neurotrophic factor (BDNF) expression.

**Materials and Methods:**

We used ovariectomized (OVX) female rats. The exercise program was mild-moderate treadmill training for 4 weeks. 72 hr SD was achieved using the multiple platform method and the spatial learning and memory parameters have been evaluated by the Morris water maze (MWM) test. The levels of studied genes were quantified by real-time PCR.

**Results:**

SD down-regulated pri-miR-1b, miR-1b (*P*˂0.05), and BDNF mRNA (*P*˂0.01) in the hippocampus. Furthermore, female rats under exercise conditions showed significant up-regulation of the miR-1b and BDNF mRNA (*P*˂0.001). In addition, miR-1b positively correlated with cognitive function (*P*˂0.05) and BDNF mRNA (*P*˂0.01).

**Conclusion:**

Our data demonstrated that regular treadmill exercise could reverse the down-regulation of hippocampal miR-1b, which has a probable role in the SD-induced cognitive impairment.

## Introduction

The positive role of physical activity on various brain functions has been demonstrated in the recent reports. Cognitive function, personality, and neurodegenerative disease have been positively affected by physical exercise (1-4). In addition, it has been shown that physical exercise, one of the most potent nonpharmacological treatments, can improve SD associated deficits such as learning and memory impairment (5, 6) and positively modulate some of the cognition-related molecules including brain-derived neurotrophic factor (BDNF) (7), PKA, and CaMKII (6). It has been shown that physical activity can regulate many gene expressions by epigenetic alterations (8).

Sleep has a vital role in normal biological functions such as thermoregulation, immune response and cognitive function especially during memory consolidation (9, 10). A large number of reports demonstrated that sleep deprivation (SD) has negative impact on the cognitive functions such as emotional memory (11), working memory (12) and hippocampus-related memory (7, 13-15). Sleep loss can also alter the protein and gene expression of the brain, which are known as the sleep regulatory molecules including NMDA and AMPA receptors, BDNF, CREB, and c-fos (16). 

 The hypothesis that SD would lead to concomitant changes in the brain microRNAs (miRNAs) expression comes from the ability of miRNAs to regulate mRNA expression. miRNA microarray analysis also determined that fifty miRNAs were changed after 8 hr SD (17). miRNAs are short and endogenous noncoding molecules which are able to affect the translation process through degradation or repression of target message RNAs (mRNAs) (18, 19). They also were used as the regulatory elements in the plasticity-related mRNAs expression (20, 21). A crucial role of miRNAs in the cognitive and neuronal functions has been reported by knocking out of the Dicer gene in the mice (22-26). 

It has been also demonstrated that BDNF, the most abundant neurotrophin in the hippocampus, is mostly associated with the hippocampus-related and cognitive functions (7, 27). An *in vitro* study showed that microRNA-1b (miR-1b) can directly inhibit BDNF expression in different cell types (28). MiR-1b can regulate the Schwann cell functions through suppressing BDNF expression in peripheral nerve injury (29). In addition, the miR-1 levels were changed after different types of exercise in the muscles and heart (30). Evaluation of the level of hippocampal miR-1b and the relation of this miRNA with learning, memory, and BDNF in the SD and exercise conditions were the main goals of this study. 

## Materials and Methods

***Animals***

We utilized Wistar female rats (weighing 200–250 g), which were kept in a standard condition (24 ± 1 °C; 12 hr light-dark schedule) (n = 8 for each group as estimated by G^*^Power software). The rats could freely obtain food and water supplies. We had five groups in this study: Control, SD, sham surgery, exercised (E), and sleep-deprived and exercised rats (E/SD). Kerman Neuroscience Research Center’s Ethics Committee approved all protocols and procedures (ethics code: KNRC-94-46) (7).

***Ovariectomy surgery***

All female rats were anesthetized using a mixture of ketamine (60 mg/kg, IP) and xylazine (10 mg/kg, IP) for all operations. All were ovariectomized under aseptic conditions. Then, all ovariectomized (OVX) female rats were housed in a recovery room for one month (31).

***Treadmill training protocol***

The animals in the exercised groups were subjected to forced running for four weeks (in the light cycle from 8:00 to 14:00). An electric shock (0.3 mA) was administered when the rats stopped training. Before the beginning of the exercise procedure, the rats habituated to the new treadmill condition for 30 min in 2 days. The mild-moderate training that gradually enhanced in speed and duration during 4 weeks was used and involved the following steps: the first and second week at 10 m/min speed for 30 min, then the time of running exercise was increased by 15 min after a week (third and fourth weeks at speed of 15 m/min). The animals rested for 5 min after every 15 min during each session (6).

***Sleep deprivation procedure***

A multiple platform set was used to induce SD, which has been explained in the previous report (7). In this experiment, the rats were sleep-deprived for 72 hr and SD induction was performed 24 hr after the last exercise period in the E/SD group. The standard conditions of temperature and light cycle were maintained during this period (32).

***Behavioral task***

Spatial learning and memory have been evaluated using the Morris water maze (MWM) test (32). It was performed 30 min after the SD paradigm during the light cycle (between 8:30 and 12:00). Training (acquisition) phase in the MWM test took approximately 3 hr. The experimental chamber (160 cm × 80 cm × 40 cm) was filled with cleaned water. Four equal quadrants were considered in the MWM pool. A submerged platform was placed near the water surface (1.5 cm) in the target quadrant of the experimental pool. A video camera connected to computer software (Noldus Ethovision ® system, version 6, USA) recorded all traces. The rats could find a submerged platform within 60 sec in four trials, which was repeated in three blocks in the acquisition phase. The animals rested on the platform for 30 sec after finding the platform. The rats were again released into the pool after resting for the next trial. There were 4 different releasing points for each rat. In each trial, latency and distance measurements were considered as a parameter of spatial learning. A probe phase was carried out 2 hr after the acquisition phase. Each rat swam for 60 sec in the probe phase. We analyzed the duration and distance of swimming in the target quadrant as the parameters of spatial memory.

***Molecular assay***

Atmospheric CO_2_ was used to anesthetize female rats for molecular experiments 24 hr after the behavioral experiments. Both hippocampi were immediately dissected on a cold surface as stated in the Paxinos and Watson’s anatomical atlas and placed in -80 ^°^C until molecular experiments.

***RNA isolation***

The rnx-plus kit was utilized to extract total hippocampal RNA (SinaClon, Iran), according to manufacturer’s instruction and afterward, the isolated RNA was dissolved in DEPC treated water. All isolated samples were quantitated by a NanoDrop-1000 UV–vis spectrophotometer (Midland, ON, Canada) and a value of ~2.0 for optical density (OD) 260/280 nm ratios was considered as pure for RNA samples. Furthermore, the electrophoresis of isolated RNA was performed in 1.5% gel of agarose.

**Figure 1:**
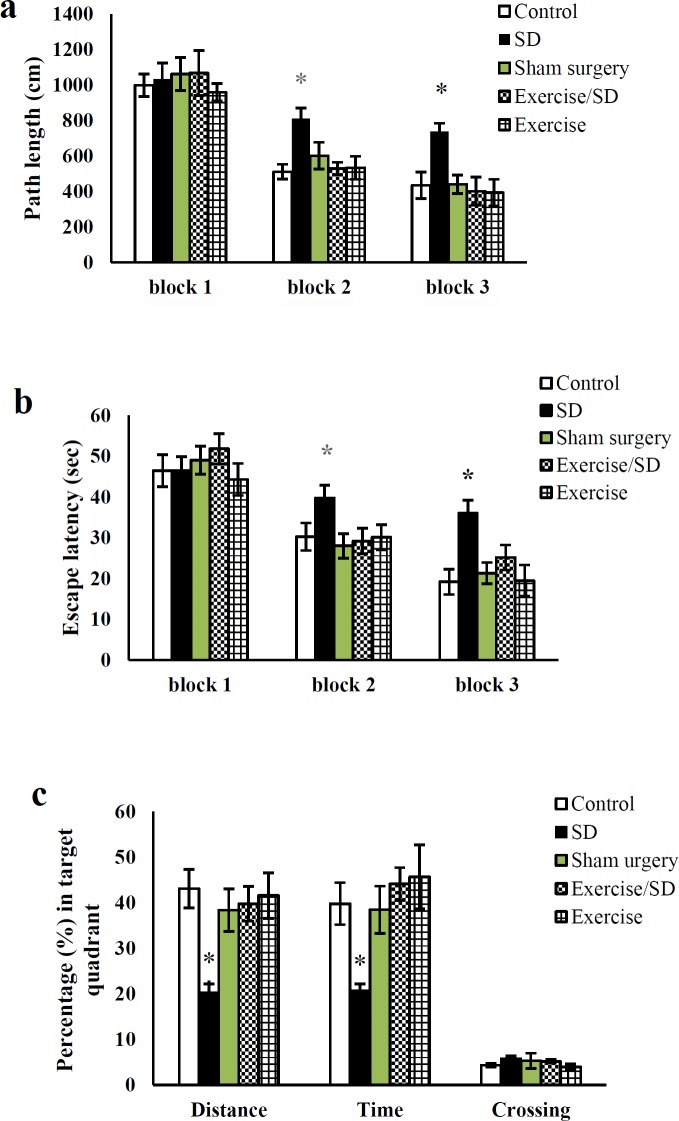
Effect of treadmill exercise and sleep deprivation (SD) on spatial learning and memory in all studied groups (a) Path length in the learning phase. (b) Escape latency in the acquisition phase. (c) The percentage of time, distance, and crossing in the target quadrant of the probe phase. Mean±SEM was measured for all groups (n= 8 rats per group) and * *P*<0.05 was considered as a significant level

**Figure 2:**
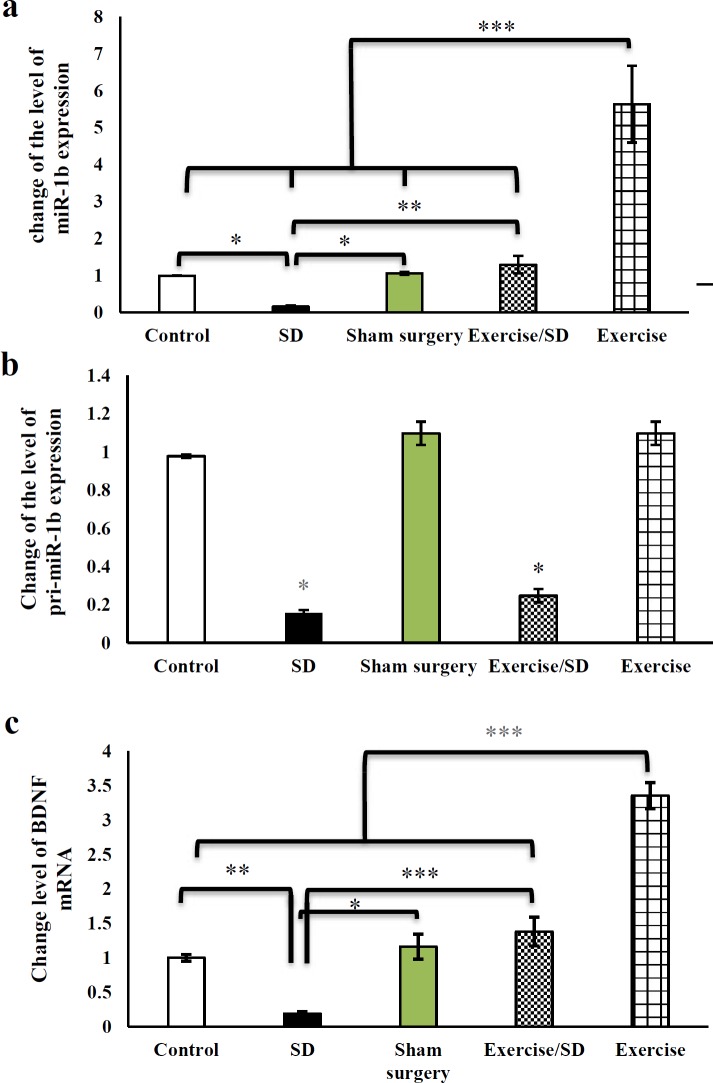
Effect of regular exercise and SD on the studied genes expression levels. (a) The miR-1b expression level in the hippocampus after treatment with SD and treadmill running. (b) Level of pri-miR-1b expression after exercise running and SD. (c) BDNF mRNA levels in all experimental groups. * *P*˂0.05, ** *P*˂0.01, and *** *P*˂0.001 were considered as significant levels

**Figure 3:**
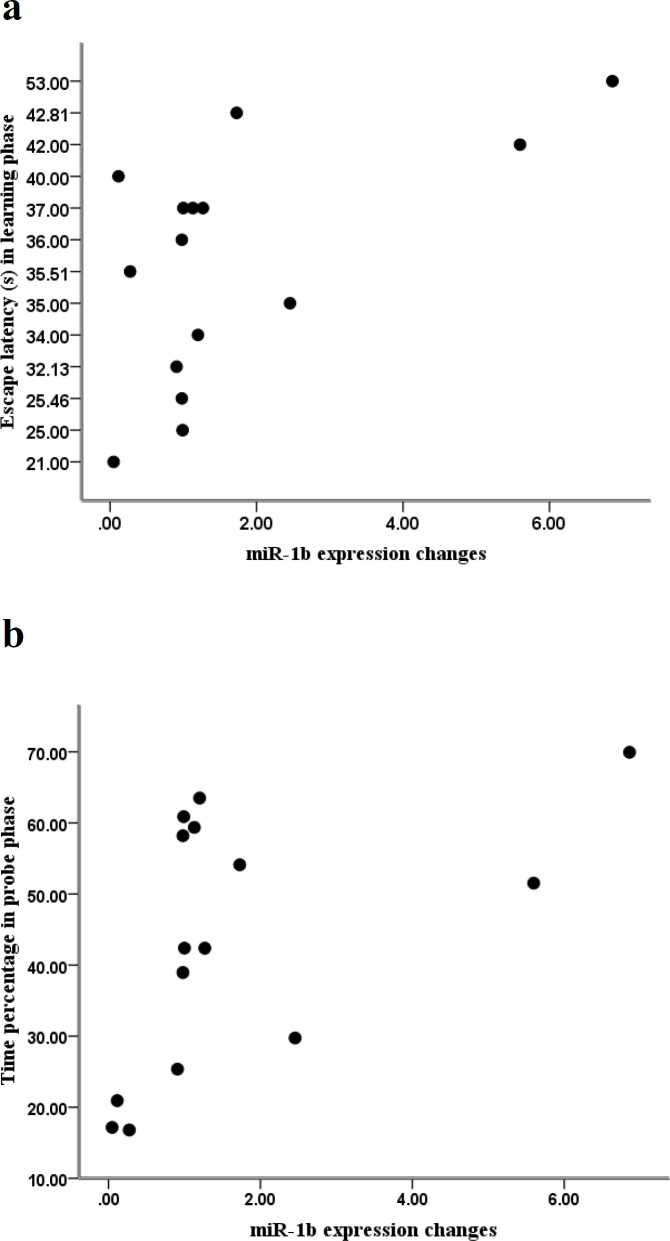
Relationship between behavioral parameters and miR-1b expression changes in the hippocampus. (A) Correlation between miR- 1b expression and escape latency in the acquisition phase. (B) Correlation between miR-1b expression and the percentage of time in the probe phase

**Figure 4:**
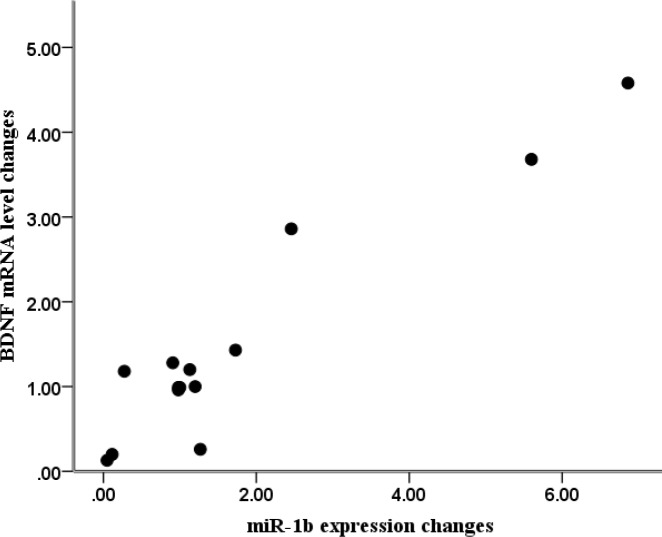
Correlation between miR-1b expression and BDNF mRNA level in the hippocampus

***Real-time PCR assay***

RT-PCR was utilized to evaluate the miR-1b, pri-miR-1b, and BDNF expression levels using ParsGenome’s miRNA amplification Kit (33). Briefly, the single-strand cDNA synthesizes of BDNF and β-actin (reference gene) was carried out using the oligo-dT primer and M-MuLV enzyme. The primers of real-time PCR were as follow: BDNF-forward: 5′-CAG CAG CAG CTG CGT GCG TGT-3′, BDNF-reverse: 5′- AGC AGT GCG TTG TAC GCC CAG C -3′; and β-actin-forward: 5′-CGC AGT GGA AGT CAG GCT TG-3′, β-actin-reverse: 5′-GGC ATA GCG TAC ACA GCG TC-3′. Due to the small size of miR-1b, pri-miR-1b, and U6 (reference gene) we couldn’t measure the level of these genes by ordinary real-time PCR assay. So, we utilized a poly (t) adaptor real-time PCR assay that especially invoked to evaluate miRNAs level. (34). The extracted RNAs were elongated by poly (A) polymerase enzyme (PAP) in the first step. The reaction was achieved in a total volume of 20 μl including 1.5 μg of RNA with 0.4 μl of PAP, 2 μl of 10x buffer, 1 μl of ATP at the temperature of 37 ^°^C for 20–30 min. After that, 5 μl of the above samples were mixed with 5x buffer, dNTP, 0.3-0.4 μl M-MuLV enzyme, and 0.3 μl of RNase inhibitor (Ribolock). The tubes were placed at 42 ^°^C for 60 min and 80 ^°^C for 3 min (to inactivate the RT enzyme). 2X SYBR Green master mix of PCR (Parstoos, Iran), cDNA products, specific primers (ParsGenome, Iran), and DEPC treated water were used to perform real-time PCR. The data analysis was performed by comparing miR-1b, pri-miR-1b, and BDNF with internal control genes (U6 and β-actin) expression level for each sample (35).

***Statistical analysis***

Group data in behavioral experiments are stated as Mean±SEM (standard error of mean). ANOVA (analysis of variance)-Turkey’s method was used to compare statistically, and *P*-values less than 0.05 were considered significant.

## Results

***The behavioral parameters in exercise and SD condition***

The analysis of repeated measures determined that the swimming distance and time in the acquisition phase of the SD group were significantly increased in block 2 (811.9±57.77 vs 510.91±42.12; *P*=0.04 and 39.77±3.12 vs 30.25±3.39; *P*=0.01, see Figures 1a and 1b, respectively) and block 3 (739.56 ± 45.31 vs 434.39 ±75.24; *P*=0.04 and 36.02±3.16 vs 19.18±3.12; *P*=0.03, see Figures 1a and 1b, respectively) as compared to the control group. The probe phase data indicated that there was memory impairment in the SD group as shown by the reduction in their distance percentage (43.07±4.23 vs 39.76±3.8; *P*=0.02, Figure 1c) and the escape latency percentage (39.8±4.6 vs 44.11±3.61; *P*=0.04, Figure 1c). It was clear that the ability of the SD group in the acquisition and probe phases was significantly improved by treadmill training.

***The hippocampal miR-1b level***

The hippocampal miR-1b levels were significantly down-regulated in the SD rats as compared to the control and sham surgery groups (*P*<0.05, Figure 2A). Four-week treadmill running significantly improved the levels of miR-1b in the SD as compared to the E/SD group (*P*<0.01, Figure 2A). MiR-1b in the exercised rats was significantly enhanced, in comparison to those of the other groups (control, SD, sham surgery, and E/SD) (*P*<0.001, Figure 2A).

***The hippocampal pri-miR-1b level***

 The pri-miR-1b levels significantly decreased in SD and E/SD groups as compared to those of the control rats(*P*<0.005) (Figure 2b) while in exercised and sham surgery groups no significant expression changes were detectable *P*<0.005 Figure 2b).

***The BDNF level of the hippocampus***

As shown in Figure 2c, the levels of BDNF in the SD rats were lower compared with the control rats (*P*<0.001 Four-week treadmill exercise elevated the level of this mRNA in the SD animals (*P*<0.001 he levels of BDNF mRNA in exercised rats were increased significantly as compared with the other groups (*P*<0.001(.

***The correlation between miR-1b expression and behavioral parameters***

Pearson correlation analysis determined that the level of miR-1b has a positive correlation with escape latency in the learning phase (r=0.688, *P*<0.001 Figure 3a). The results indicated that miR-1b expression also shows a positive correlation with the percentage of time in the probe phase (r=0.587, *P*<0.005, Figure 3b). The other behavioral parameters didn’t correlate to the miR-1b expression changes (data not shown).

***The correlation between miR-1b expression and BDNF mRNA level***

We investigated the relationship between hippocampal miR-1b and BDNF levels and the results showed that miR-1b expression changes are positively correlated to the BDNF mRNA changes (r=0.932, *P*<0.001 Figure 4(.

## Discussion

Evaluation of the hippocampal miR-1b levels in the exercise and SD conditions was the most important aim of this study. We used ovariectomized female (OVX) rats in our experiment to clear the ovarian hormones effects on the miR-1b expression changes in the SD condition. The molecular data showed that 72 hr SD can decrease the miR-1b level, which was ameliorated by four weeks of treadmill exercise. In addition, regular exercise also induced hippocampal miR-1b up-regulation in the of OVX rats.

In this study, the OVX group was more susceptible to the SD effects on the miR-1b expression than intact female animals (sham surgery). Interestingly, our previous lab experiment has demonstrated these negative SD effects in OVX animals in different paradigms such as cognitive functions and BDNF levels (7, 32). Recently, it has been indicated that there was a low level of miR-1 in the liver and midbrain of mice, which was undetectable by Northern analysis (36). 

It has been shown that a number of miRNAs are found outside of the cells, including in several body fluids (37). MiR-1, one of the circulating miRNAs, can be transported through blood (38). Because miR-1 has been demonstrated to be up-regulated in the heart and muscles and also function in other tissue (39), we suggested that miR-1b may be secreted into the blood and functioned in the neurons of the hippocampus. To confirm whether changes of miR-1b levels were due to changes in the endogenous biogenesis of neurons or transported from other tissues, the levels of pri-miR-1 were also detected in the hippocampus of all rats.

 In this study, we found that the level of pri-miR-1b decreased in the hippocampus of SD and E/SD groups, while miR-1b down-regulated only in the SD group. These results suggested that SD leads to the down-regulation of hippocampal miR-1b by reducing the endogenous biogenesis of this miRNA not transporting from the other organs. This finding is consistent with other findings demonstrating that 6 hr SD has no effect on the expression of miR-1b in the mouse model hearts (40). In addition, treadmill running can reverse the down-regulation of miR-1b induced by SD, but it doesn’t have any effect on the pri-miR-1b expression level. In fact, running exercise improved the deleterious effect of SD on miR-b expression by transporting it from the other organs. These results are in agreement with a previous study of forced running that increases the miR-1b level in the mice muscles (41). It has also been reported that miR-1 is up-regulated in the muscles after 60 min of endurance exercise (42). However, it has been reported that cycling reduces the miR-1 expression level in the skeletal muscles. The differences in training type, length, and intensity and experiment may lead to these contradictory outcomes.

Surprisingly, in this study, a positive correlation between hippocampal miR-1b levels and some behavioral parameters (escape latency in the learning phase and percentage time in probe tests) revealed that down-regulation of this miRNA also can be related to the SD-induced cognitive dysfunction. Learning and memory are two of the hippocampus-dependent functions that are affected by SD. In addition, a positive correlation between miR-1b and BDNF mRNA levels demonstrates the relation of this miRNA with cognitive function. Cardiac up-regulation of miR-1 induced cognitive impairment, which may be correlated with the down-regulation of hippocampal BDNF level (39). It has been also determined that there is a regulatory loop between BDNF and this miRNA, which was interrupted with the deletion of BDNF in the mouse models (43). In this study, down-regulation of hippocampal miR-1b in sleep-deprived rats disturbed this regulatory loop and may be a consequence of reduced BDNF level, which must be clarified in the next studies.

## Conclusion

We determined a significant down-regulation of hippocampal miR-1b after 72 hr SD that was attenuated by 4-week treadmill exercise. In addition, the hippocampal pri-miR-1b level showed that up-regulation of miR-1b in the exercised group might have occurred due to its transportation from the other organs, but the down-regulation of this miRNA in the SD group originates from the reducing endogenous biogenesis of miR-1b in the hippocampal neurons.
